# Transport and Mobility Segregation in Urban Spaces

**DOI:** 10.23889/ijpds.v8i3.2268

**Published:** 2023-09-11

**Authors:** Nandini Iyer, Ronaldo Menezes, Hugo Barbosa

**Affiliations:** 1 University of Exeter; 2 University of Ceará

## Introduction & Background

Public transportation is one of many factors that influence the level of disadvantage in a city. By facilitating movement within urban areas, transit systems can democratise accessibility to resources, while also fostering social integration among individuals from different areas and sociodemographic backgrounds. Conversely, inequalities in transport services can hinder individuals from fulfilling their travel demands. In this work, we explore socioeconomic segregation in cities from the perspective of their transit systems and how it intersects with segregation levels on a residential and employment level.

## Objectives & Approach

In our analyses, we combine socioeconomic data from the 2020 American Community Survey with amenity visitation patterns from anonymised mobile phone traces, provided by SafeGraph, to estimate the mobility flows between areas (i.e., Census Block Groups - CBGs) in a given city. We define a CBG's segregation level using the Index of Concentration at the Extremes, which ranges from -1 to 1, reflecting extreme concentration of individuals from low and high income groups, respectively. Moreover, we retrieve General Transit Feed Specification and OpenStreetMap data to construct transit-pedestrian networks for various US cities.

## Relevance to Digital Footprints

We leverage digital footprints, in the form of mobility flows between CBGs, to estimate the socioeconomic composition of different public transport routes within a city. By combining digital footprints with the respective economic breakdowns of trip origins, and transit-pedestrian networks, we can develop a better understanding of how segregated individuals are throughout various contexts of urban life.

## Results

While segregation still exists in the transport and amenity dimensions, our findings suggest that individuals are exposed to the highest magnitudes of segregation in the residential dimension, with amenity and transit segregation allowing for potential avenues for reducing experiential segregation. However, we observe that the transit service in many cities hinders individuals in low-income neighbourhoods from accessing areas characterised by more affluent socioeconomic backgrounds.

## Conclusions & Implications

These results underscore research that reveals how mobility patterns in neighbourhoods with a high concentration of underprivileged demographics, be it immigrant or ethnic minorities, tend to have more constrained activity spaces than their privileged counterparts. Although it is unclear whether mobility patterns are influenced by segregation levels of neighbourhoods, it is apparent that by limiting exposure to different types of neighbourhoods, transit systems impose constraints on the activity space and urban experience of individuals, namely those without access to personal vehicles. We highlight the benefit of analysing segregation as a spatio-temporal experience rather than a static variable, showing how mobility is used as a tool to try and overcome residential segregation. Moreover, identifying inequalities within transit systems is the first step in providing improved transit service, particularly to individuals from especially vulnerable demographics. Ultimately, by identifying how transit infrastructure may perpetuate segregation, we pursue the first of many steps to re-imagining transport as a point of inclusion within the urban realm.

